# Rad eating disorder education for student dietitians

**DOI:** 10.1186/2050-2974-3-S1-P21

**Published:** 2015-11-23

**Authors:** Elesa Crowley, Deanne Harris, Leanne Brown

**Affiliations:** 1 Hunter New England Local Health District, Newcastle, Australia; 2 The University of Newcastle Department of Rural Health, Newcastle, Australia

## 

Student dietitians are often very interested in developing skills in the assessment and management of clients with eating disorders. This may be difficult; clients often decline student participation in their care due to the sensitive nature of their condition. Nourishing Networks, a self-directed health professional learning program, was developed to enhance clinician knowledge and management skills for individuals with disordered eating and assist with the formation of local networks of support. This program was adapted and piloted for dietetic students attending placement at the University of Newcastle Department of Rural Health Tamworth. The aim of the program was to enhance the students', knowledge and skills in the management of eating disorders. Students were offered the opportunity to participate in the 10 week program. Results showed all students furthered their knowledge, skills and attitudes towards eating disorders and were better able to determine appropriate treatment plans. This program allowed students to gain tools for working with clients that they would not otherwise be exposed to in their undergraduate degree and associated placements. Future plans include the presentation of an Eating Disorder Interprofessional Learning Module for nursing, medical and allied health students and subsequently offering this 10 week program to these students.

